# Elevated Infant Mortality Rate among Dutch Oral Cleft Cases: A Retrospective Analysis from 1997 to 2011

**DOI:** 10.3389/fsurg.2014.00048

**Published:** 2014-12-04

**Authors:** Daan P. F. van Nunen, Marie-José H. van den Boogaard, J. Peter W. Don Griot, Mike Rüttermann, Lars T. van der Veken, Corstiaan C. Breugem

**Affiliations:** ^1^Division of Plastic and Reconstructive Surgery, University Medical Center Utrecht, Utrecht, Netherlands; ^2^Department of Medical Genetics, University Medical Center Utrecht, Utrecht, Netherlands; ^3^Department of Plastic, Reconstructive and Hand Surgery, Vrije Universiteit University Medical Center Amsterdam, Amsterdam, Netherlands; ^4^Department of Plastic Surgery, University Medical Center Groningen, Groningen, Netherlands

**Keywords:** cleft lip, cleft palate, Robin sequence, Pierre Robin syndrome, infant mortality, epidemiology, Netherlands

## Abstract

**Objectives:** First, to determine the infant mortality rate (IMR) for Dutch patients with isolated oral clefts (OC) as well as for patients with clefts seen in association with other malformations. Second, to conduct a similar analysis per cleft type: cleft lip with or without cleft palate (CP), CP (including Robin sequence). Third, to examine the underlying causes of death.

**Material and Methods:** A retrospective review of the charts of patients with OC born in the period 1997–2011 and treated in three regional cleft centers in the Netherlands.

**Results:** One thousand five hundred thirty patients with OC were born during the study period and treated in the cleft centers. The overall IMR for all clefts was 2.09%, significantly higher than the general Dutch IMR of 0.45%. In a subanalysis per cleft type, the IMRs were 1.22, 1.38, 2.45, and 3.62% for cleft lip, cleft lip with CP, CP, and Robin sequence, respectively. The mortality rates for *isolated* OC did not differ significantly from the general Dutch rate. Causes of death were congenital malformations of the heart in 40.6%, airway/lungs in 15.6%, nervous system in 15.6%, infectious disease in 12.5%, and other or unknown in 15.6%.

**Conclusion:** The elevated IMR observed in Dutch patients with OC is almost exclusively caused by associated congenital malformations. After diagnosis of an oral cleft an in-depth medical examination and a consult by the pediatrician and clinical geneticist is imperative to instigate the appropriate medical management.

## Introduction

Oral clefts (OC) in the form of cleft palate (CP) and cleft lip with or without CP (CLP, CL) are among the most common congenital malformations with a prevalence of 2.7 per 1000 live births in the Netherlands (1). In addition to isolated occurrence, OCs are frequently associated with other congenital malformations and recognized syndromes (2). Historical studies demonstrated an increased mortality rate amidst infants with OCs in conjunction with further malformations (3–5). In a recent meta-analysis, Carlson et al. confirmed these findings and established an odds of dying in the first year of life of 9.47 [95% confidence interval (CI), 6.15–14.56] for all infants with OCs relative to the general population (6). However, after excluding patients with additional malformations in a subanalysis, the odds ratio remained a significant 2.07 (95% CI 1.39–3.09) (6), which implies that even infants with an isolated OC are at an increased risk of early death.

Previously, no figures have been published on the mortality rates of infants with OCs in the Netherlands. The objective of the present study was to obtain the Dutch infant mortality rate (IMR) for patients with OCs, including those with additional congenital malformations or syndromes. In further analyses, the IMRs for isolated OCs and for distinctive cleft types are presented. Special attention is given to the IMR in patients with CP in the context of Robin sequence.

## Materials and Methods

In the Netherlands, all surviving children born with an OC are referred to 1 of the 15 regional OC teams, which offer multidisciplinary treatment according to local protocols. Members of each treatment team belong to the Dutch Association for Cleft Palate and Craniofacial Anomalies (*NVSCA*), which manages a national register of orofacial clefts and craniofacial anomalies to facilitate epidemiological and clinical research. The register was launched in 1997 and since then the clinical characteristics of every child seen by one of the teams were entered into the register at the moment of the first consultation. A peculiar characteristic of the register is that it does not allow for updates later than 24 h after creating a patient record, which may preclude it from containing complete information on all congenital comorbidities and/or genetic disorders. In-depth information on the register is found in the overview by Luijsterburg and Vermeij-Keers (7).

A retrospective epidemiological study was conducted into the IMR among live-born children with OCs treated by three Dutch regional cleft centers: the VU University Medical Center Amsterdam (*VUmc*), the University Medical Center Groningen (*UMCG*), and the University Medical Center Utrecht (*UMCU*). Prior approval for this study was obtained from the Institutional Review Boards. Dutch law did not require parental informed consent, since patients were not subject to investigational actions. Infants with OCs born in the period 1997–2011 and under treatment in the three cleft centers were identified through their registration with the NVSCA. The date of birth and type of OC were acquired from the national register for all infants that were not adopted and who were residing in the Netherlands at the moment of registration. Subsequently, at each cleft center a review of the medical records was performed (A) to verify the presence of concomitant congenital malformations and syndromes in the documentation of the consulted clinical geneticist, (B) to assess the mortality status, and (C) to obtain the cause of death. The mortality status was cross-checked with the Dutch Municipal Personal Records Database and no disagreements were found. The overall IMR in the Netherlands during 1997–2011 was provided by the Dutch Central Statistics Office (8).

Subsequently, the IMR for all OC cases was calculated with IBM SPSS Statistics 20.0 (IBM Inc., New York, NY, USA). In a subanalysis, separate IMRs were derived for each type of OC as well as for isolated clefts. The results were compared to the overall IMR in the Netherlands using Odds ratios. A *P*-value of <0.05 was considered statistically significant.

## Results

### Infant mortality

From 1997 to 2011 5401 infants with OCs were registered with the NVSCA by all regional OC teams in the Netherlands, of which 1420 (26.3%) had CL, 2144 (39.7%) CLP, and 1837 (34.0%) CP. The three cleft centers participating in this study were responsible for 1530 registrations (or 28.3% of the total) consisting of 411 (26.9%) infants with CL, 589 (38.5%) with CLP, and 530 (34.6%) infants with CP (Table 1, panel A). Associated congenital malformations or syndromes were present in 26.1% of all OC cases, ranging from 8.8% in CL to 50.9% in CP. A total of 32 deaths were recorded before the age of 1 year, resulting in an IMR of 2.09%, which was significantly higher than the overall IMR of 0.45% witnessed in the Netherlands during this period. Among cases of isolated OC the IMR did not differ from the overall national level (Table 1, panel B).

**Table 1:** **Infant mortality rates**.

(A) All OCs	*N*	% Of total	IMR (%)	Odds^a^	95% CI	*P*
CL	411	26.9	1.22	2.73	1.13–6.59	0.025
CLP	589	38.5	2.38	5.39	3.17–9.17	0.000
CP	530	34.6	2.45	5.57	3.21–9.67	0.000
RS subpopulation	138	–	3.62	8.32	3.41–20.35	0.000
Total	1530	100	2.09	4.73	3.33–6.73	0.000

**(B) Isolated OCs**	***N***	**% Of incl.**	**IMR (%)**	**Odds^a^**	**95% CI**	***P***

CL	336	91.2	0.27	0.59	0.08–4.22	0.601
CLP	496	84.2	0.20	0.45	0.06–3.18	0.422
CP	260	49.1	0.77	1.72	0.43–6.91	0.446
Total	1131	73.9	0.35	0.79	0.29–2.10	0.632

*^a^Relative to the general Dutch IMR of 0.45% (1997–2011)*.

For patients with CL, CLP, and CP (including associated malformations), IMRs of 1.22, 2.38, and 2.45% were observed, all significantly higher than the national IMR (Table 1, panel A). Isolated cases of CL, CLP, and CP had IMRs insignificantly different from the overall IMR prevailing in the Netherlands. A sizable subgroup of 26.0% of the CP patient population suffered from Robin sequence, characterized by micro- or retrognathia, glossoptosis, and obstructive respiratory distress. Patients with Robin sequence, either in isolation or with additional malformations, had a significantly elevated IMR of 3.62%.

### Causes of death

Congenital heart defects were reported as the principal cause of death among infants with OCs and were responsible for around of 40% of deaths across cleft types (Table 2, panel A). Other leading causes of death were malformations of the airways and lungs and malformations of the brain and nervous system, each resulting in over 15% of deaths. Infectious disease and sepsis caused one-eighth of infant deaths, generally in the presence of extensive congenital malformations. Another 12.5% of deaths were ascribed to sudden infant death syndrome (SIDS) or were of unknown origin. In cases of isolated OCs only four deaths were reported, one due to infectious disease, and three to unknown causes or SIDS. Additional details on all deceased infants are provided in the Supplementary Material (Table S1 in Supplementary Material).

**Table 2:** **Causes of death**.

Cause of death	Type of OC							
	CL		CLP		CP		Total	
	*N*	%	*N*	%	*N*	%	*N*	%
**(A) All OCs**								
Congenital heart defect	2	40.0	6	42.9	5	38.5	13	40.6
Congenital malformation airways/lungs			2	14.3	3	23.1	5	15.6
Congenital malformation brain/nervous system	1	20.0	3	21.4	1	7.7	5	15.6
Infectious			2	14.3	2	15.4	4	12.5
Other					1	7.7	1	3.1
Unknown/sudden infant death syndrome	2	40.0	1	7.1	1	7.7	4	12.5
Total	5	100.0	14	100	13	100	32	100
**(B) Isolated OCs**							
Infectious					1	50.0	1	25.0
Unknown/sudden infant death syndrome	1	100.0	1	100.0	1	50.0	3	75.0
Total	1	100.0	1	100.0	2	100.0	4	100.0

## Discussion

This study demonstrates that Dutch infants with OCs had an increased risk of dying in the first year of life compared to the general infant population in the Netherlands. However, the heightened odds of dying of 4.73 (95% CI 3.73–6.73) were lower than the 9.47 (95% CI 6.15–14.56) reported in the meta-analysis by Carlson et al. (6) for OC patient populations. Explanations for this difference include the influence of older studies with high-mortality rates in the meta-analysis by Carlson et al., advances in prenatal diagnostics, disparities in the standards of care and patient monitoring and, finally, probable differences in the distribution and definition of associated malformations and causes of death.

For cases of isolated OCs no elevated IMR was found in this study, in contrast to the increased odds of 2.07 (95% CI 1.39–3.09) obtained by Carlson et al. Three of the seven original studies in this meta-analysis had similar insignificantly raised mortality rates for isolated OCs (9–11). The examination into the causes of the 32 deaths reported in the Dutch patient cohort showed that the raised IMR for *all* OC patients was primarily the result of congenital malformations other than orofacial clefts. It is therefore conceivable that the heightened IMR for *isolated* OCs found by Carlson et al., resulted from an underdiagnosis of possibly fatal associated malformations or syndromes in several (older) studies included in their meta-analysis.

Again, in the subanalysis per cleft type increased odds of infant death were noted for the cohorts that included patients with other congenital malformations, but not in the cohorts that contained only isolated cases. The IMRs for CL, CLP, and CP were slightly lower than the varying rates reported in earlier studies (5, 9–12). The reasons for these discrepancies remain unknown, as further details on the patient populations and causes of death were not provided in these papers. Patients with CP in the context of Robin sequence, either in isolation or with associated malformations, had the greatest odds of dying in the first year of life. Interestingly, only one of these patients died due to upper airway obstruction. The IMR of 3.62% for all patients with Robin sequence is in line with experiences in the literature (13–16).

This study is the first to offer insights in the mortality rates of infants with OCs in the Netherlands and has several merits. First, the use of data by three tertiary pediatric teaching hospitals allowed for a proper patient follow-up in the first year of life with few referrals to other centers. Second, the identification of patients through the NVSCA cleft registry ensured the collection of an unbiased sample of the Dutch OC patient population, as affected infants receive their first consult at the closest regional cleft center, which also completes the registration irrespective of later referral elsewhere. Third, in contrast to earlier studies from abroad, the causes of death were examined showing the fatal influence of concomitant congenital malformations.

Limitations of this study include the possibility that a small number of infants with severe congenital disease were inadvertently excluded due to lack of involvement of the cleft team prior to their early deaths. Second, formal autopsies were few in number, which forced the authors to rely on the causes of death as reported by the medical teams responsible for the deceased patients at the time of death. Third, given the retrospective nature of the study, subtle associated malformations or syndromes could have been underreported (17).

The main clinical relevance of our findings lies in the recognition that the elevated mortality in infants with OCs is predominantly caused by associated congenital malformations and that a significant proportion of these patients are affected by them. Therefore, if a cleft is diagnosed prior to or after birth, an in-depth medical examination and a consultation by both pediatrician and clinical geneticist are imperative. Furthermore, the results of this study are applicable in pre- and postnatal counseling sessions with parents. An OC may be part of spectrum of congenital anomalies or a named syndrome that carries an elevated mortality risk (9). However, if the cleft appears to be an isolated phenomenon after sufficient investigations, the parents could be provided with the comforting message that their child has no significantly increased odds of an early demise.

## Conflict of Interest Statement

The authors declare that the research was conducted in the absence of any commercial or financial relationships that could be construed as a potential conflict of interest.

## Supplementary Material

The Supplementary Material for this article can be found online at http://www.frontiersin.org/Journal/10.3389/fsurg.2014.00048/abstract
