# Supplementary material for: Elevated Infant Mortality Rate among Dutch Oral Cleft Cases: A Retrospective Analysis from 1997 to 2011
Source: Front Surg. 2014 Dec 4;1:48. doi: 10.3389/fsurg.2014.00048 (PMC4286963; doi:10.3389/fsurg.2014.00048)
Supplement: Supplementary file 1 [file Table1.PDF]

APPENDIX

Table A1: Characteristics of patients deceased during the first year of life

No.	Type	Chromosomal / DNA anomalies[A]	Chromosomal / DNA anomalies[B]	Syndrome	Other congenital malformations	Cause of death
1.	CL	arr [hg19] 1p36.33p36.31 (833,900-6,049,871)x1, 19q13.42q13.43 (55,859,227-59,092,714)x3	Deletion chr.1p36	1p36 deletion syndrome	VSD[C], cerebral anomalies	Status epilepticus
2.	CL	-	-	*Isolated*	-	Sudden infant death syndrome
3.	CL	-	-	No identified syndrome	MCADD[D], spina bifida, Arnold Chiari malformation	Unknown
4.	CL	47,XY,+18	Trisomy 18	Edwards syndrome	VSD[C], PDA[E], omphalocele, etc.	Heart failure
5.	CL	Mutation *CHD7* gene c. [1495C>T]p.[Q499*]	Mutation *CHD7* gene	CHARGE syndrome	CHARGE spectrum, extensive	Heart failure
6.	CLP	47,XX,+21	Trisomy 21	Down syndrome	Holoprosencephaly, AVSD[C], diabetes insipidus, etc.	Heart failure
7.	CLP	46,XY.arr 8q24.13(126,150,171-126,218,003)x1	Deletion chr. 8q24.13	CHARGE syndrome	CHARGE spectrum, extensive	Heart failure
8.	CLP	-	-	No identified syndrome	Arthrogryposis multiplex congenita, heart defects	Sepsis
9.	CLP	-	-	*Neonatal abstinence syndrome (methadone)*	-	Sudden infant death syndrome
10.	CLP	-	-	No identified syndrome	Spina bifida aperta	Respiratory depression due to hydrocephalus
11.	CLP	-	-	No identified syndrome	Left innominate artery, complex structural liver defects	Compression of trachea by left innominate artery
12.	CLP	-	Deletion chr. 17q21.31	Microdeletion syndrome del 17q21.31	Complete AVSD[C]	Heart failure
13.	CLP	47,XY,+13	Trisomy 13	Patau syndrome	Tetralogy of Fallot, trigonocephaly, aplasia cutis, etc.	Heart failure
14.	CLP	47,XX,+13	Trisomy 13	Patau syndrome	Microcephaly, aplasia cutis, extensive dysmorphic features, etc.	Heart failure
15.	CLP	-	Duplication chr.18q11.2	No identified syndrome	Mitral valve atresia, hypoplastic left ventricle / asc. aorta, hydrocephalus, etc.	Heart failure
16.	CLP	-	Duplication chr.14q12	No identified syndrome	Hydrocephalus, PDA[E], anorectal malformation, hydronephrosis, etc.	Respiratory depression due to hydrocephalus

17.	CLP	-	-	No identified syndrome	Extensive dysmorphic features, cerebral malformations	Respiratory failure due to central vocal cord paralysis
18.	CLP	-	-	Goltz-Gorlin syndrome	Cutaneous laesions, skeletal deformations	Sepsis
19.	CLP	-	-	No identified syndrome	Tracheomalacia, right desc. aorta, colonic atresia, Peter's anomaly, hypothyroidism	Respiratory failure due to tracheomalacia
20.	CP	46,XY.ish del(22)(q11.2q11.2) dn	Deletion chr. 22q11.2 de novo	Velo-cardio-facial syndrome	ASD-II[C], malalignment VSD[C], BAV[F], hypoplastic aortic arch with coarctation, etc.	Heart failure
21.	CP	46,XX,del(4)(q31.3q35) dn	Deletion chr. 4q31.3-q35 (de novo)	4q31q35 deletion syndrome	Robin seq., Coarctation of aorta, VSDs[C], microcephaly, right renal hypoplasia, urachal cyst, etc.	Sepsis
22.	CP	-	-	No identified syndrome	Robin seq., spondyloepiphyseal dysplasia, CTEV[G], etc.	Heart failure
23.	CP	-	-	Probable Yunis-Varon syndrome	Robin seq.; extensive cardiac, renal, cerebral and skeletal malformations	Upper airway obstruction due to glossoptosis
24.	CP	-	Inversion duplication chr 8p	No identified syndrome	Robin seq., VSD[C], cerebral malformations	Central neurogenic respiratory failure
25.	CP	-	-	*Isolated*	-	Unknown
26.	CP	-	-	No identified syndrome	Laryngotracheomalacia, omega epiglottis, malformations nervous system	Airway obstruction due to laryngotracheomalacia
27.	CP	-	-	No identified syndrome	Complete AVSD[C]	Heart failure
28.	CP	-	Unbalanced translocation chr 1;15 (duplication 15q15->qter, deletion 1q42->qter)	No identified syndrome	Robin seq.; extensive cardiac, aortic, renal and genitourinary malformations	Aspiration of stomach contents
29.	CP	-	Deletion chr.16q24.1-24.2	No identified syndrome	Laryngomalacia, non-septated lungs, capillary alveolar dysplasia, spinal malformations, retro-/micrognathia, etc.	Respiratory and circulatory failure in PPHN[H] due to lung anomalies
30.	CP	-	-	*Isolated*	-	Sepsis
31.	CP	-	-	No identified syndrome	ASD[C], VSD[C], tricuspid valve atresia	Heart failure
32.	CP	Mutation CHD7 gene c.[781del] [p.ser 261fs]	Mutation *CHD7* gene	CHARGE syndrome	CHARGE spectrum, extensive	Heart failure, necrotizing enterocolitis

A. According to nomenclature
B. Descriptive
C. Atrial / Ventricular Septum Defect
D. Medium-Chain Acyl-coenzyme A Dehydrogenase Deficiency
E. Patent Ductus Arteriosis
F. Bicuspid Aortic Valve
G. Congenital Talipes Equino Varus
H. Persistent Pulmonary Hypertension of the Newborn
